# From regeneration to immunomodulation: a 20-year global bibliometric analysis of platelet-rich plasma for osteoarthritis

**DOI:** 10.3389/fmed.2026.1790140

**Published:** 2026-03-25

**Authors:** Yuchen Wang, Hongchen Ren, Jingbo Cheng, Haicheng Tao, Mingli Feng

**Affiliations:** Department of Orthopaedic Surgery, Xuanwu Hospital, Capital Medical University, Beijing, China

**Keywords:** bibliometrics, inflammation, osteoarthritis, platelet-rich plasma, research hotspots

## Abstract

**Background:**

Osteoarthritis (OA) is a whole-joint disease driven by complex immunopathogenic mechanisms and chronic inflammation, positioning platelet-rich plasma (PRP) as an emerging therapy of significant interest.

**Objective:**

This study conducts a bibliometric analysis to systematically map the global research landscape, hotspots, and immunological trends of PRP for the treatment of OA from 2004 to 2024.

**Methods:**

We analyzed 1,862 articles and reviews retrieved from the Web of Science Core Collection (WoSCC) for bibliometric mapping, complemented by 148 clinical trial records from PubMed for qualitative analysis. We utilized VOSviewer, CiteSpace, and the R-package bibliometrix for visual analysis of countries, institutions, authors, journals, and keywords.

**Results:**

The results demonstrate a significant growth in annual publication volume since 2012, with the United States (1,675 publications) and China (1,310 publications) being the most productive countries. The Rizzoli Orthopaedic Institute, the Hospital for Special Surgery, and Rush University were identified as core research institutions, while Giuseppe Filardo was the most prolific author. *The American Journal of Sports Medicine* ranked as the leading journal in both publication count and total citations. Keyword analysis identified four major research hotspots: disease targets and tissue mechanisms, pathophysiological processes, comparative clinical treatments, and research methodologies. Recent research frontiers include “macrophage polarization” and “exercise.”

**Conclusion:**

This study provides bibliometric evidence that the research landscape of PRP for OA is transitioning from a focus on tissue regeneration to immunomodulation. The field has evolved from observing clinical outcomes to unraveling the intricate immune-mediated mechanisms, particularly the regulation of macrophage phenotypes and inflammatory mediators. Future research should prioritize standardizing PRP protocols based on their immunomodulatory potential and further exploring strategies to precisely manipulate the joint inflammatory environment for therapeutic gain.

## 1 Introduction

Osteoarthritis (OA) is a prevalent chronic joint disease that represents a leading cause of disability worldwide, affecting over 500 million individuals (1, 2). It manifests across various anatomical sites with distinct epidemiological profiles; the knee is the most frequently affected joint (with a prevalence of 43% in individuals over 40 years of age), followed by the hip (7.3% in adults over 30), while OA of the shoulder and ankle (prevalence < 1%) is less common but highly debilitating (3–5). The pathogenesis of OA is driven by a complex interplay of systemic and local risk factors. At the systemic level, advanced age, female sex, and genetic predisposition increase susceptibility, while whole-body conditions like obesity, sarcopenia, and metabolic inflammation act as crucial drivers of disease progression (6). At the local joint level, biomechanical stressors—such as previous trauma, anatomical malalignment, and abnormal joint loading—serve as primary triggers for joint degeneration (5, 7). Now understood as an inflammatory disease of the entire joint organ, its pathology involves cartilage degeneration, subchondral bone alterations, and synovitis, leading to significant pain, functional impairment, and a reduced quality of life for patients (8–10). While current management strategies, such as pharmacotherapy and eventual joint replacement surgery, can alleviate symptoms, they are predominantly palliative. These treatments do not fundamentally reverse cartilage damage or promote tissue regeneration (11, 12). Consequently, there is a critical need for novel therapies that can intervene early to delay or even reverse disease progression before irreversible structural damage occurs.

Platelet-Rich Plasma (PRP) is an autologous platelet concentrate derived from a patient’s own blood (13). As a biologically active product, PRP is rich in growth factors and cytokines, giving it the potential to promote tissue regeneration, alleviate inflammation, and modulate the joint microenvironment, which makes it a promising therapy for OA (14, 15). Consequently, research in this field has expanded rapidly, with numerous studies exploring PRP’s ability to reduce pain, improve function, and delay cartilage degeneration (16–18). The proposed mechanisms include improving the intra-articular microenvironment by suppressing inflammation while promoting anabolism, and potentially exerting direct analgesic effects via cannabinoid receptors (19, 20). Despite this growing body of research, significant challenges remain. The lack of standardized protocols for PRP preparation (leading to variations in platelet and leukocyte concentrations) results in poor comparability across studies and hinders the establishment of unified clinical standards. Therefore, its precise efficacy, optimal indications, and long-term outcomes are still debated (21–23). Given these complexities, a systematic analysis of the extensive literature is urgently needed to map the field’s developmental trajectory and identify key trends.

Bibliometrics is an interdisciplinary field that uses statistical methods to analyze literature, revealing a domain’s knowledge structure, key contributors, and emerging trends, thereby providing evidence-based predictions for future research directions (24). Despite the volume of research on PRP for OA, a comprehensive bibliometric analysis of this specific intersection is lacking. Therefore, this study will use bibliometric methods and visualization tools (VOSviewer, CiteSpace, R-package bibliometrix) to analyze two decades of literature (2004–2024) using a dual-database strategy combining the Web of Science Core Collection (WoSCC) and PubMed, in accordance with the TITAN Guidelines 2025 for the declaration and use of artificial intelligence in research reporting (25).

Our analysis will identify the primary contributing countries, institutions, and authors and map their collaborative networks. By examining high-impact journals and highly-cited documents, we will highlight the field’s milestone achievements. Furthermore, through keyword and thematic evolution analyses, this study will delineate the core research hotspots and their dynamic changes over time. Ultimately, this research aims to provide a clear landscape of the field for scholars, clinicians, and policymakers, with the goal of promoting the standardization and advancement of PRP in both basic and clinical applications for OA.

## 2 Materials and methods

### 2.1 Data collection

The bibliometric analysis was primarily conducted using the WoSCC (Capital Medical University Version) due to its comprehensive metadata and suitability for citation network analysis. However, to provide a deeper insight into the specific clinical translation of PRP therapy, we employed a dual-database strategy. While WoSCC was used for visual mapping, the PubMed database was independently queried to specifically retrieve and analyze clinical trial records.

**FIGURE 1:**
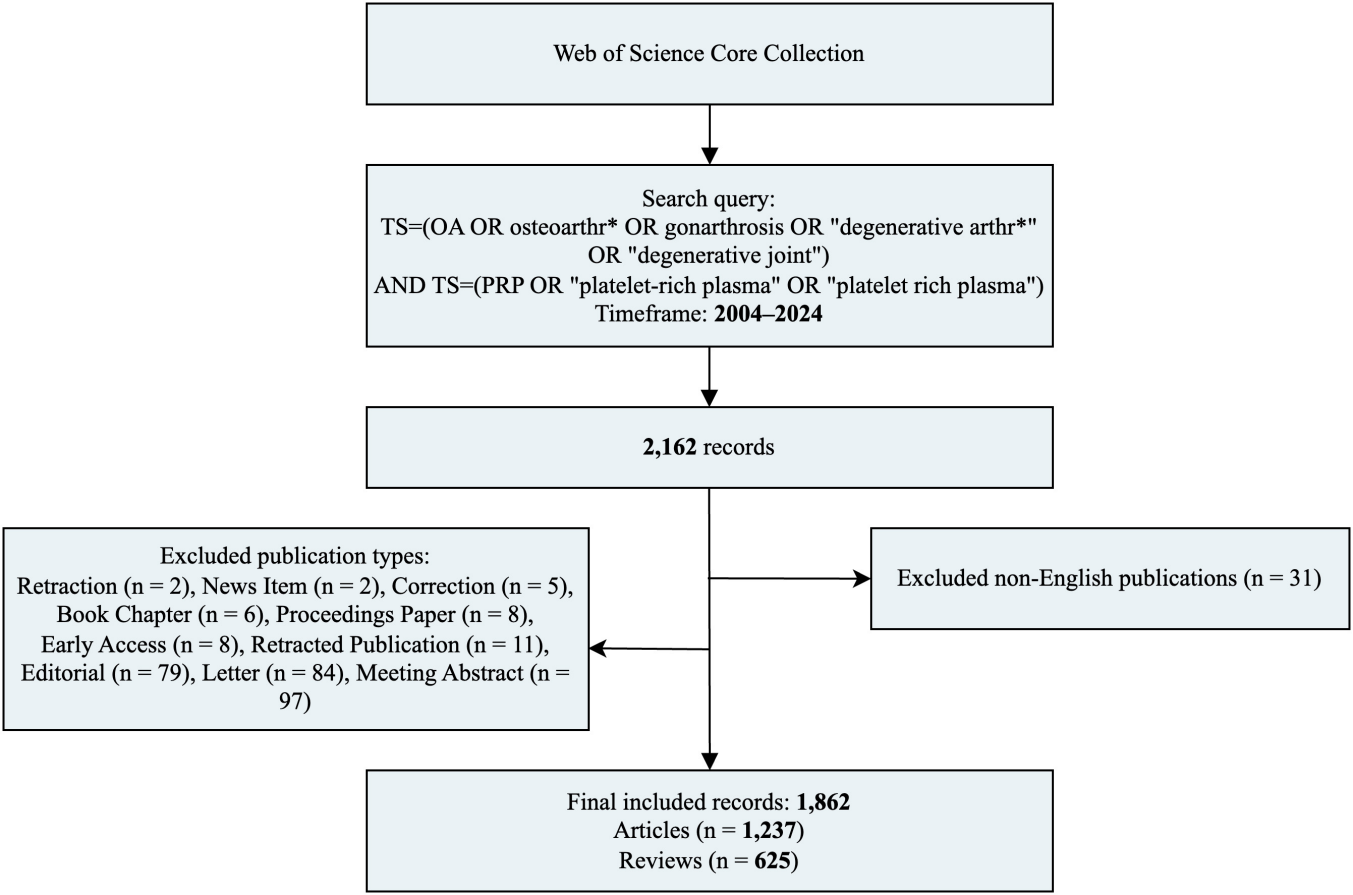
Data processing flow chart of the bibliometric analysis based on the WoSCC.

The search in WoSCC was employed: (TS = (OA OR osteoarthr* OR gonarthrosis OR “degenerative arthr*” OR “degenerative joint”) AND TS = [PRP OR “platelet-rich plasma” OR “platelet rich plasma”)]. The search was limited to the English language (LA = English), publication years ranging from 2004 to 2024 [PY = (2004–2024)], and document types restricted to Articles or Reviews [DT = (Article OR Review)]. Figure 1 illustrates the detailed literature screening process, including the number of publications at each stage. The search in PubMed was limited to “Clinical Trial” and “Randomized Controlled Trial” publication types, spanning from January 1, 2004, to December 31, 2024. The specific search string was: [“Osteoarthritis”(Mesh) OR osteoarthr*(Title/Abstract) OR OA(Title/Abstract)] AND [“Platelet-Rich Plasma”(Mesh) OR “platelet-rich plasma”(Title/Abstract) OR PRP(Title/Abstract)] AND [Clinical Trial (pt) OR Randomized Controlled Trial (pt)] AND English[la] AND 2004:2024[dp].

### 2.2 Data analysis

This study employs bibliometric methods and visualization tools to conduct quantitative and qualitative analyses of the included literature data. The purpose is to reveal the overall landscape, major contributors, collaborative patterns, research hotspots, and emerging trends in the field of OA and PRP research. The primary bibliometric analyses and network visualizations were completed using the following software tools.

VOSviewer (version 1.6.20) was used to construct and visualize co-occurrence networks of countries, institutions, authors, and keywords to identify collaborative patterns and research themes. The R-package bibliometrix (version 4.3.5) was employed for descriptive statistical analyses, such as annual publication trends and thematic evolution, while ggplot2 (version 3.5.2) was used for creating customized graphics. Finally, CiteSpace (version 6.3.1) was used to perform citation burst analysis to identify milestone achievements and research frontiers. Journal impact factors (IF) were based on the 2023 Journal Citation Reports.

The clinical trial records retrieved from PubMed were reviewed qualitatively. These records were not merged with the WoSCC dataset for bibliometric mapping but were analyzed independently to summarize current clinical application trends, treatment protocols, and outcome measures (as presented in the *Clinical Progress Analysis* section).

## 3 Results

### 3.1 Analysis of annual publication output

**FIGURE 2:**
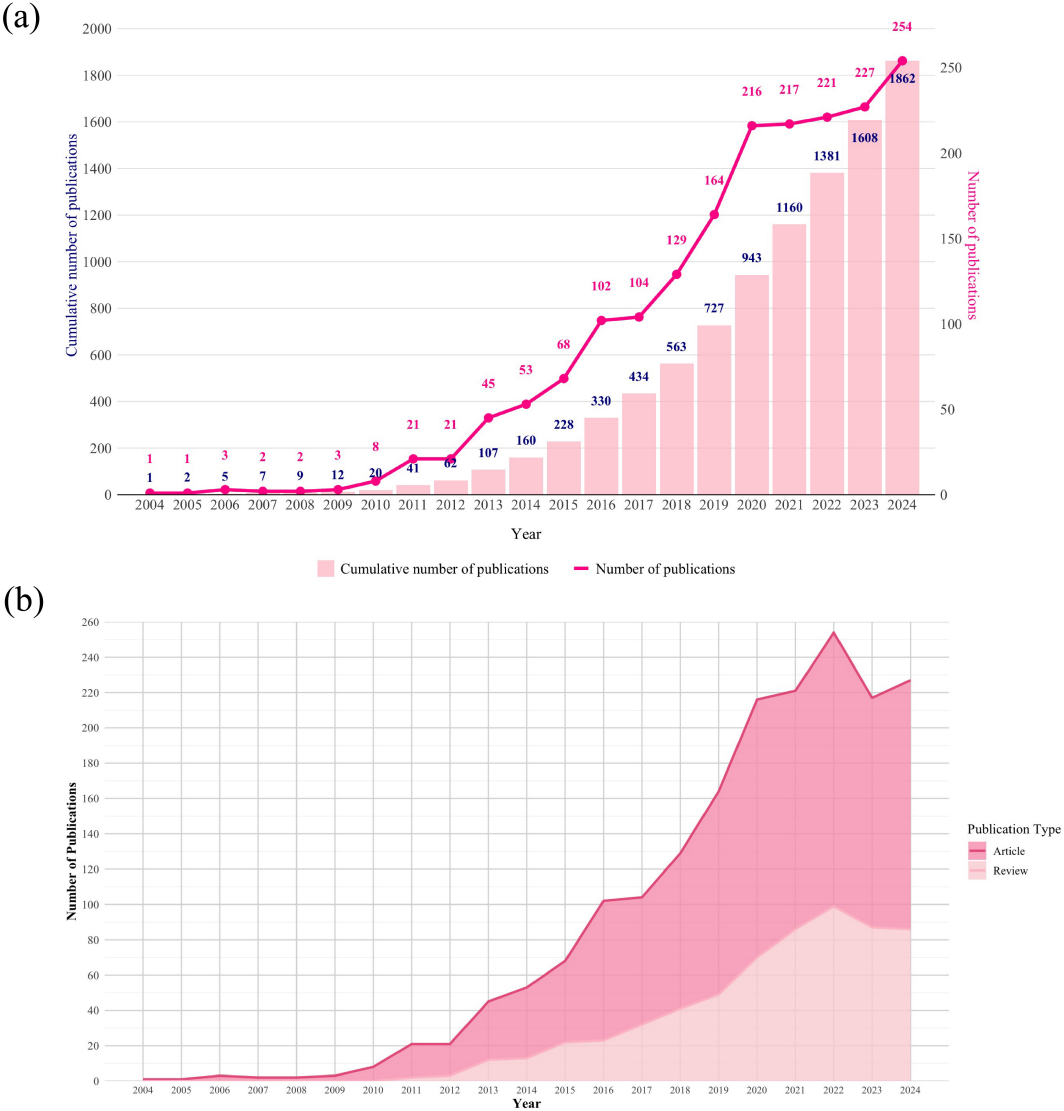
**(a)** Annual and cumulative number of publications. **(b)** Annual number of publications by type (article and review).

Our bibliometric analysis based on WoSCC included 1,862 publications on OA and PRP from 2004 to 2024. The annual publication volume has shown sustained growth over this period (Figure 2a). After an initial slow phase with fewer than 30 articles per year (2004–2011), the field entered rapid development in 2012. Output then climbed steadily, accelerated sharply around 2019, and peaked at 254 articles in 2024, demonstrating strong recent research momentum.

An analysis by document type (Figure 2b) reveals that while original “Articles” existed from 2004, their numbers only began to grow significantly after 2009. “Reviews” first appeared in 2011, signaling that the field had matured from exploratory research to a stage requiring the systematic synthesis of existing findings.

### 3.2 Analysis of major institutions and authors

**FIGURE 3:**
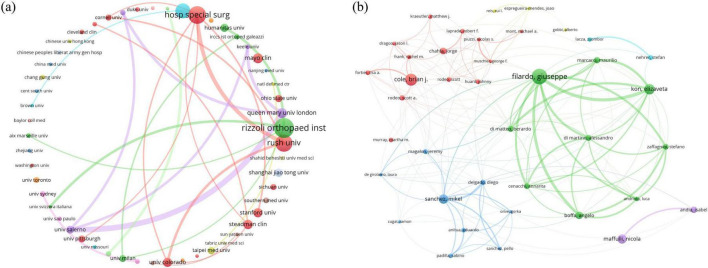
**(a)** Map of collaboration between different institutions. **(b)** Map of collaboration between authors.

Institutional collaboration analysis identified the Rizzoli Orthopaedic Institute (Italy), the Hospital for Special Surgery (United States), and Rush University (United States) as the most productive research centers (Figure 3a). The Hospital for Special Surgery anchors an extensive global network, while the Rizzoli Orthopaedic Institute serves as a key bridge connecting European and American institutions. In the author analysis, Giuseppe Filardo (Italy) was the most prolific and central scholar, leading a prominent research cluster with key collaborators like Elizaveta Kon and Alessandro Di Martino (Figure 3b).

### 3.3 Country contributions and collaboration

**FIGURE 4:**
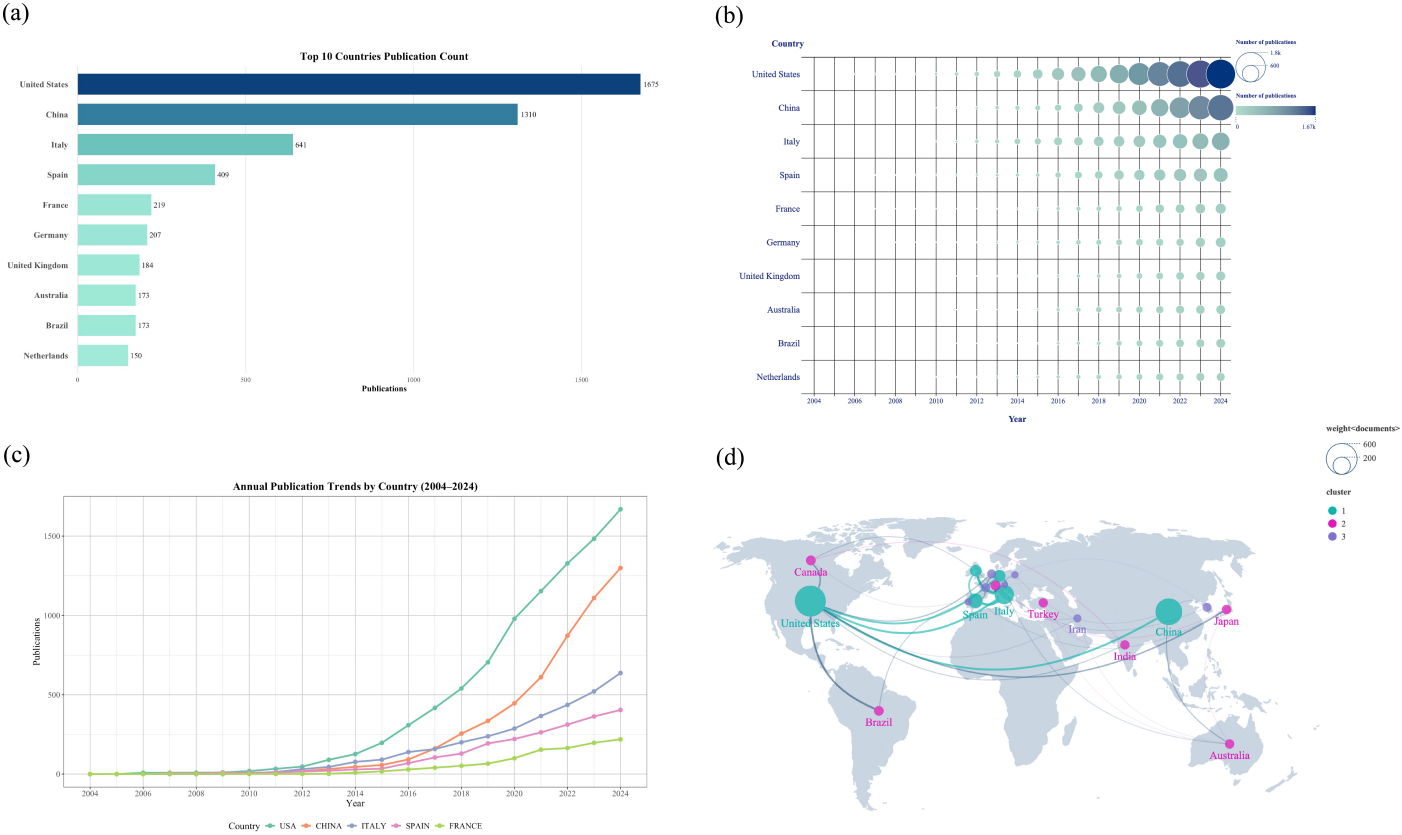
**(a)** Top 10 countries by publication count. **(b)** Annual publication output by the top 10 countries. **(c)** Annual publication trends for the top 5 most productive countries. **(d)** Map of collaboration between countries.

The United States (1,675 publications) and China (1,310) are the primary global contributors, followed by Italy (641) and Spain (409) (Figure 4a). Annual output from the top countries has grown steadily, particularly in the last decade. Notably, while the United States has maintained its lead, China has shown remarkable growth, accelerating after 2012 to surpass Italy for the second rank around 2017 (Figures 4b,c).

In terms of international collaboration, the United States, China, and a European cluster led by Italy form the core of the global network, with prominent ties between the United States and the other two hubs (Figure 4d). A closer look at collaboration strength reveals the Italy-United Kingdom partnership as the most active (value: 29). The China- United States collaboration is also highly significant, ranking fourth globally and highlighting the strong connection between the field’s most productive nations (Table 1).

**TABLE 1:** Top 10 country pairs with closest global collaboration.

Rank	Country pair	Collaboration value
1	Italy-United Kingdom	29
2	Italy-Switzerland	28
3	Italy-Spain	27
4	China-United States	22
5	Italy-United States	22
6	Germany-Italy	22
7	Germany-United States	21
8	Brazil-United States	20
9	Spain-United Kingdom	18
10	Canada-United States	15

### 3.4 Journal analysis summary

**FIGURE 5:**
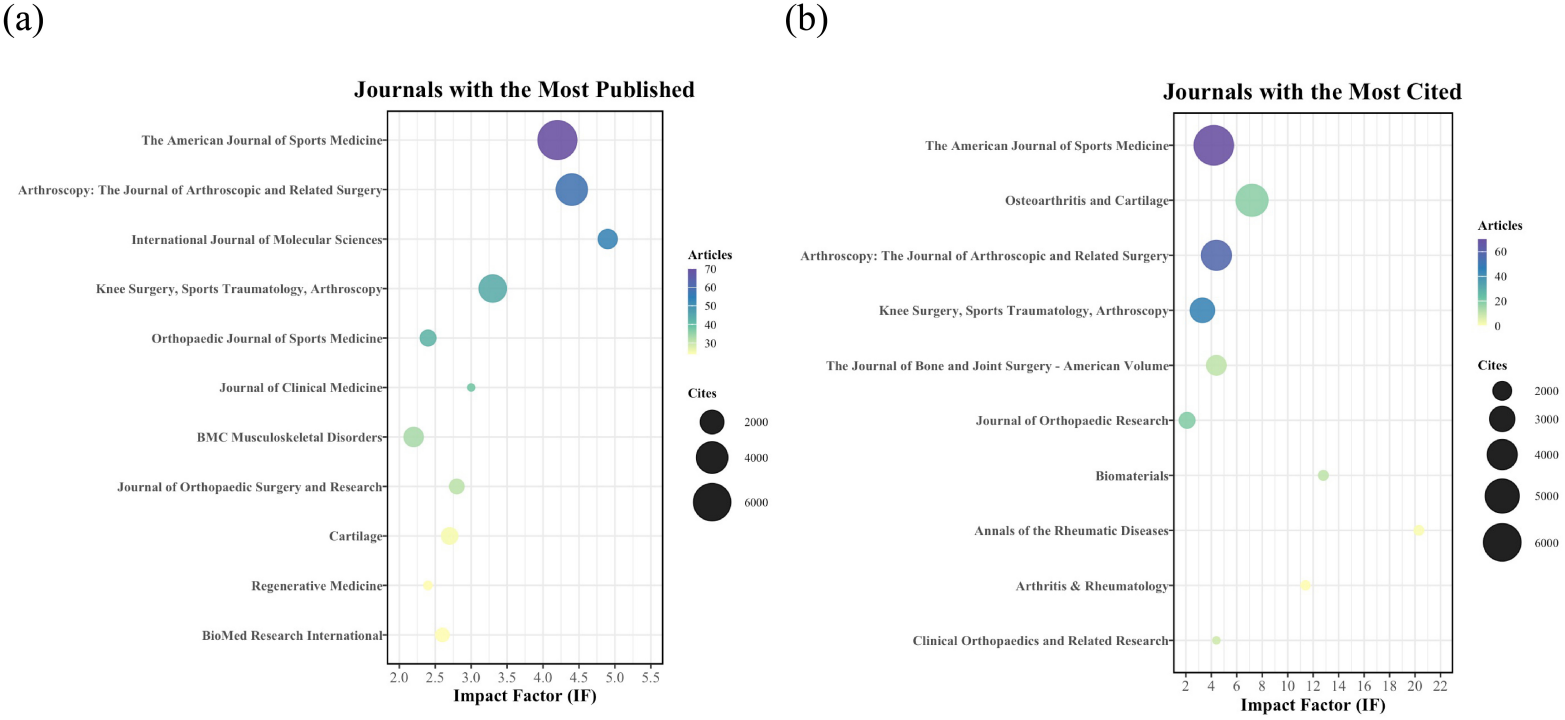
**(a)** Journal with the largest number of articles published. **(b)** Journals with the largest number of citations.

*The American Journal of Sports Medicine* stands out as the core journal in this field, ranking first in both publication volume (70 articles) and total citations (6,623). *Arthroscopy: The Journal of Arthroscopic and Related Surgery* follows, ranking second in publications (58) and third in citations (3,957). These two journals represent the primary publication venues. In contrast, some high-volume journals, such as the *International Journal of Molecular Sciences* (53 articles), have accrued substantially fewer citations, indicating a gap between publication quantity and field-specific impact (Figure 5a and Table 2).

**TABLE 2:** Top 10 journals with the most published.

Sources	Cites	IF (2023)	Articles
The American Journal of Sports Medicine	6,623	4.2	70
Arthroscopy: The Journal of Arthroscopic and Related Surgery	3,957	4.4	58
International Journal of Molecular Sciences	1,200	4.9	53
Knee Surgery, Sports Traumatology, Arthroscopy	2,842	3.3	44
Orthopaedic Journal of Sports Medicine	768	2.4	42
Journal of Clinical Medicine	302	3	38
BMC Musculoskeletal Disorders	1,228	2.2	33
Journal of Orthopaedic Surgery and Research	650	2.8	31
Cartilage	843	2.7	25
BioMed Research International	558	2.6	24
Regenerative Medicine	313	2.4	24

An analysis by total citations highlights another type of influential journal: those with high impact despite low publication volume. *Osteoarthritis and Cartilage* exemplifies this trend, ranking second for total citations (4,444) from only 18 articles, supported by a high IF (7.2). Furthermore, the presence of top-tier specialty journals like *Annals of the Rheumatic Diseases* (IF 20.3, 1 article) and *Biomaterials* (IF 12.8, 10 articles) among the most-cited sources demonstrates that the topic has sufficient quality to attract attention from premier, interdisciplinary platforms (Figure 5b and Table 3).

**TABLE 3:** Top 10 journals with the most cited.

Sources	Cites	IF (2023)	Articles
The American Journal of Sports Medicine	6,623	4.2	70
Osteoarthritis and Cartilage	4,444	7.2	18
Arthroscopy: The Journal of Arthroscopic and Related Surgery	3,957	4.4	58
Knee Surgery, Sports Traumatology, Arthroscopy	2,842	3.3	44
The Journal of Bone and Joint Surgery-American Volume	2,112	4.4	10
Journal of Orthopedic Research	1,701	2.1	19
Biomaterials	1,365	12.8	10
Annals of the Rheumatic Diseases	1,360	20.3	1
Arthritis & Rheumatology	1,351	11.4	0
Clinical Orthopedics and Related Research	1,332	4.4	8

### 3.5 Citation bursts

**FIGURE 6:**
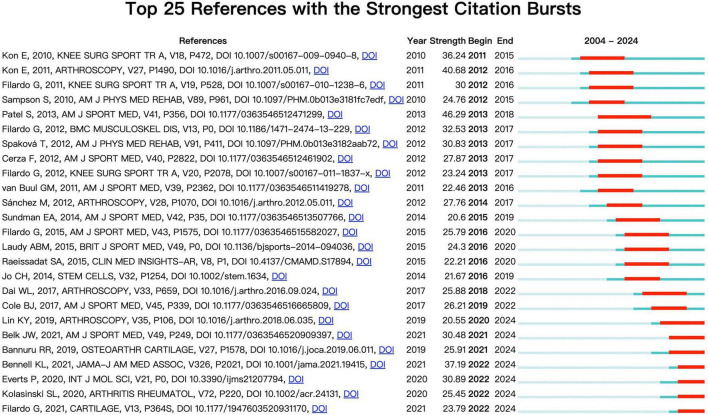
Top 25 references with the strongest citation bursts in PRP for OA treatment.

Citation Burst Analysis is an effective method for identifying literature that have experienced a sharp increase in citations over a specific period. We conducted a citation burst analysis using CiteSpace to identify key literature representing research breakthroughs or focal points. The analysis identified the 25 references with the strongest citation bursts between 2004 and 2024 (Figure 6). Figure 6 lists the burst strength, begin year, end year, and duration for each of these references.

The information for the three references with the highest citation burst strength is as follows: (1) “Treatment With Platelet-Rich Plasma Is More Effective Than Placebo for Knee Osteoarthritis” (Strength: 46.28); (2) “Platelet-Rich Plasma Intra-Articular Injection Versus Hyaluronic Acid Viscosupplementation as Treatments for Cartilage Pathology: From Early Degeneration to Osteoarthritis” (Strength: 40.68); (3) “Effect of Intra-articular Platelet-Rich Plasma vs. Placebo Injection on Pain and Medial Tibial Cartilage Volume in Patients With Knee Osteoarthritis” (Strength: 37.19). Furthermore, the titles of the three most cutting-edge citation bursts include: (1) “Platelet-Rich Plasma: New Performance Understandings and Therapeutic Considerations in 2020;” (2) “2019 American College of Rheumatology/Arthritis Foundation Guideline for the Management of Osteoarthritis of the Hand, Hip, and Knee;” (3) “PRP Injections for the Treatment of Knee Osteoarthritis: A Meta-Analysis of Randomized Controlled Trials.” Collectively, these findings indicate that while comparing PRP with other intra-articular therapies was an early hotspot, the research frontier has shifted to more rigorous, multi-faceted investigations into PRP’s true efficacy.

### 3.6 Keyword clusters and evolution

Keyword analysis is an effective method for revealing the research hotspots within a specific academic field. In this study, we utilized VOSviewer software to extract keywords from the included literature, identifying a total of 4,615 keywords. Table 4 lists the 20 most frequently occurring keywords and their specific frequencies. Among them, “hyaluronic acid” ranked first with a frequency of 665 occurrences, closely followed by “knee osteoarthritis” (636 occurrences), “cartilage” (459 occurrences), and “intra-articular injection” (437 occurrences). These high-frequency terms provide a preliminary outline of the core themes and focal points in the research field of OA and PRP. Regarding anatomical sites, keyword analysis demonstrates that knee osteoarthritis is the predominant focus of current PRP research. As shown in Table 4, knee-related terms occurred 930 times, vastly outnumbering hip-related terms (141 times) and other joints like the ankle or shoulder. Thus, the reviewed literature primarily evaluates PRP efficacy within the knee joint.

**TABLE 4:** Top 20 keywords.

Rank	Words	Occurrences	Rank	Words	Occurrences
1	Hyaluronic acid	665	11	Efficacy	287
2	Knee osteoarthritis	636	12	Management	200
3	Cartilage	459	13	Regenerative medicine	198
4	Intra-articular injection	437	14	Therapies	169
5	Mesenchymal stem cells	405	15	Stem cells	145
6	Growth factors	366	16	Hip	141
7	Injection	354	17	Repair	139
8	Double-blind	347	18	Placebo	138
9	Chronic pain	301	19	Meta-analysis	132
10	Knee	294	20	Chondrocytes	129

**FIGURE 7:**
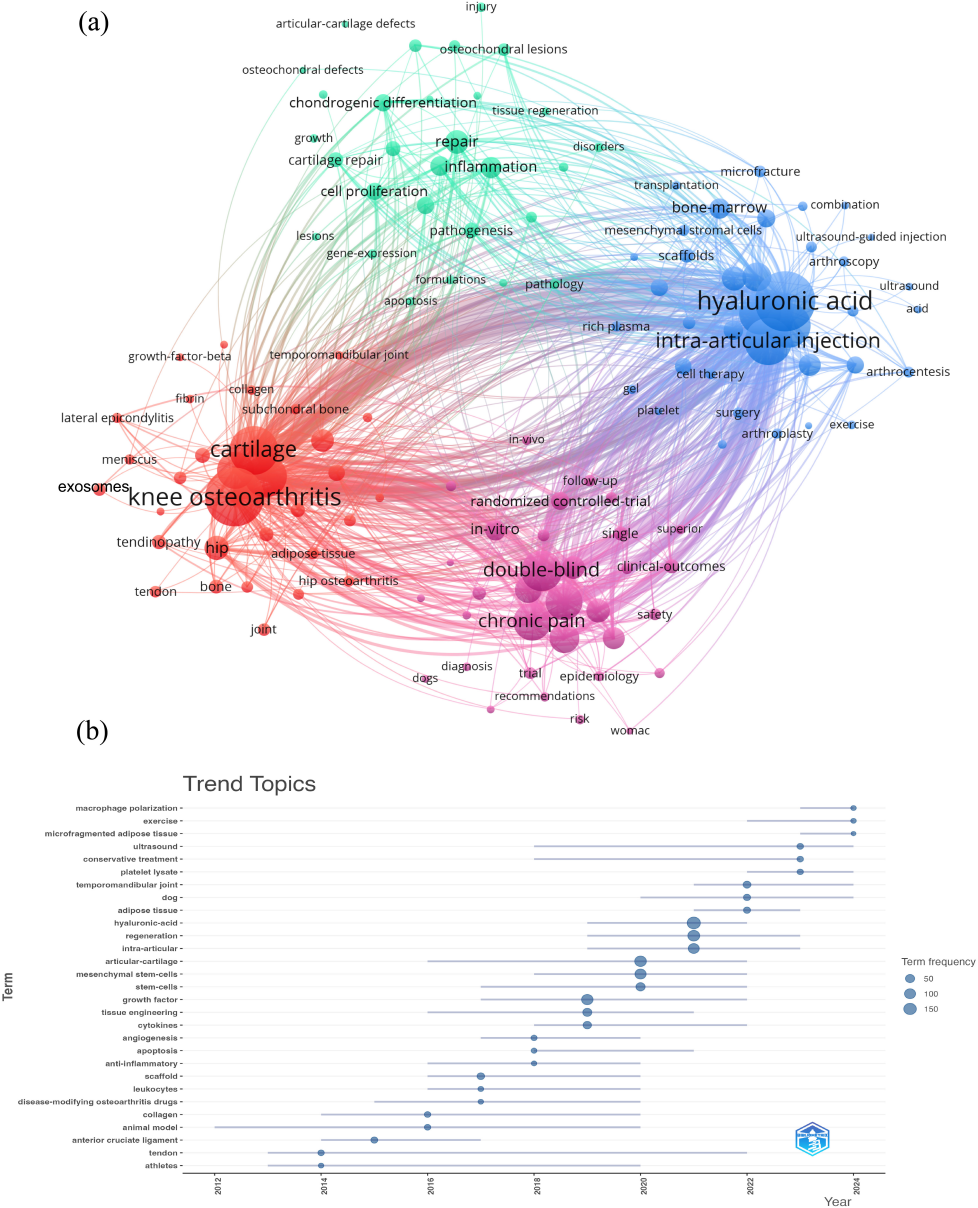
**(a)** Keyword clustering map. **(b)** Trend topics on PRP and OA.

To further explore the relational structure among various themes, we used VOSviewer to construct a co-occurrence network map of 131 keywords with a co-occurrence frequency of at least 20. This resulted in the formation of four primary keyword clusters, as shown in Figure 7a. (1) Targets in disease and tissue mechanisms (red cluster): This cluster contains 32 keywords. Core keywords include representative diseases or affected sites such as “knee osteoarthritis,” “hip osteoarthritis,” “lateral epicondylitis,” and “temporomandibular joint,” as well as key pathological tissues like “cartilage,” “meniscus,” and “subchondral bone.” It also includes important research targets at the cellular and molecular levels, such as “chondrocytes,” “exosomes,” and “transforming growth factor-beta.” (2) Pathophysiological processes and conditions (green cluster): This cluster is composed of 29 keywords, which primarily represent pathological states related to OA, the processes of disease development, and key biological links that PRP treatment may intervene in. Representative terms include “inflammation,” “cartilage repair,” “cartilage defects,” and “chondrogenesis.” (3) OA treatments compared or combined with PRP (blue cluster): This is the largest cluster, containing 37 keywords. The keywords

in this group are mainly focused on the comparison of PRP with other therapeutic modalities in OA treatment and the exploration of combination strategies. Core terms include “hyaluronic acid,” “intra-articular injection,” “mesenchymal stem cells,” “bone marrow,” and “surgery.” (4) Research methods and evaluation (pink cluster): This cluster contains 33 keywords. The keywords are primarily focused on the design of clinical research in the OA and PRP field, its methodological quality, and the standards for efficacy evaluation. Representative terms include “double-blind,” “randomized controlled trial,” and “chronic pain.”

To delineate the temporal evolution of research themes, a dynamic thematic evolution map was constructed using the R-package bibliometrix (Figure 7b). The analysis revealed the primary research keywords prevalent during distinct periods. In the 2012–2016 period, the main thematic keywords included “tendon,” “anterior cruciate ligament,” “animal model,” and “collagen.” Subsequently, from 2016 to 2019, terms related to the exploration of therapeutic mechanisms and tissue engineering, such as “tissue engineering,” “scaffold,” “cytokines,” “angiogenesis,” “apoptosis,” and “anti-inflammatory,” emerged as new high-frequency topics. Between 2019 and 2022, the research focus narrowed to the application of PRP in articular cartilage regeneration, specifically exploring its combination with cellular therapies like stem cells, and its comparison or co-application with existing treatments such as hyaluronic acid. Correspondingly, keywords including “mesenchymal stem cells,” “articular cartilage,” “regeneration,” and “hyaluronic acid” demonstrated a significant increase in frequency and prominence. In the most recent period, 2022–2024, the latest emerging high-frequency themes included “macrophage polarization,” “exercise,” “microfragmented adipose tissue,” and “platelet lysate.”

### 3.7 Clinical progress analysis

Using the search strategy described in the Data Collection section, a total of 148 clinical trials were identified in the PubMed database and exported in NBIB format. These studies can be broadly categorized into six main research themes: (1) comparative efficacy analysis of PRP versus placebo or standard therapies (e.g., hyaluronic acid) for OA; (2) evaluation of combination therapies utilizing PRP with other therapeutic agents or rehabilitation strategies; (3) exploration of PRP applications across diverse anatomical joints, including the knee, hip, ankle, and temporomandibular joints; (4) investigation into formulation optimization and dosing protocols, such as leukocyte content and injection frequency; (5) assessment of PRP as an adjunct to surgical interventions (e.g., anterior cruciate ligament reconstruction or meniscectomy); and (6) study of biological mechanisms and structural changes via imaging and biomarker analysis.

## 4 Discussion

### 4.1 Global research landscape

Over the past two decades (2004–2024), the annual publication volume in the research field of PRP for OA has climbed rapidly, exhibiting exponential growth particularly after 2012, which indicates a surging academic interest in this area.

An analysis of institutions and authors reveals that research efforts are not evenly distributed but are highly concentrated, showing a distinct “clustering effect.” A few top-tier institutions, notably the Rizzoli Orthopaedic Institute (Italy), the Hospital for Special Surgery (United States), and Rush University (United States), function as both the primary research producers and crucial global collaboration hubs. At the individual level, Giuseppe Filardo from Italy is the most prolific scholar, leading a tightly-knit network of other significant contributors. This small number of highly productive and collaborative institutions and teams constitutes the core force of the field. Through the extensive networks they have built, they collectively drive knowledge innovation and serve as an essential knowledge base for other researchers.

The United States is the primary global contributor to this field, with 1,675 publications. Its sustained leadership stems from foundational work by American pioneers, including Leland Kaiser’s concept of “regenerative medicine” in 1992 and Robert Marx’s crucial quantification of PRP standards in 1998 (26, 27). This historical advantage, combined with top research institutions like the Hospital for Special Surgery and Rush University, has solidified its leading position. China ranks second with 1,310 publications. Despite a later start, it has demonstrated remarkable growth, accelerating after 2012 and surpassing Italy in 2017. This surge is strongly linked to immense domestic clinical demand, driven by a large and rapidly aging population that provides abundant case resources for research (28). This is coupled with significant national research support, as addressing health issues related to aging has become a key government priority (29). The global collaboration network is centered around three core hubs: the United States, China, and the European cluster. Within Europe, Italy has emerged as the most important regional core, driven by its close collaborations with countries like the United Kingdom, Switzerland, and Spain. Italy’s pivotal role is further underscored by the fact that it is home to the field’s most significant contributors: the Rizzoli Orthopaedic Institute and the top-ranking scholar, Giuseppe Filardo. A noteworthy phenomenon is that the United States and China, as the two most prolific countries in this field, are also each other’s closest collaborators. Moreover, as shown in Table 1, collaborations involving the United States occupy multiple positions in the top 10 (with strong ties to other major countries like Germany, Brazil, and Canada), which further underscores its central role in the global collaboration network.

At the journal level, *The American Journal of Sports Medicine* and *Arthroscopy: The Journal of Arthroscopic and Related Surgery* have published the most research, establishing sports medicine and arthroscopic surgery as the core disciplines for this topic. A secondary cluster of journals, including the *International Journal of Molecular Sciences*, *BioMed Research International*, and *Regenerative Medicine*, forms another key publishing outlet, focusing on the intersection of orthopedic research and molecular biology. While publication volume is an important metric, it doesn’t equate to a journal’s influence or prestige. For instance, although the *International Journal of Molecular Sciences* has a high publication output in this field, its papers have accumulated significantly fewer citations than those in *The American Journal of Sports Medicine* and *Arthroscopy: The Journal of Arthroscopic and Related Surgery*, highlighting a disparity between quantity and impact. When evaluating journal influence by total citations, *The American Journal of Sports Medicine* is the undisputed leader, serving as the primary platform for both publication volume and impact. In contrast, *Osteoarthritis and Cartilage* exemplifies a different type of authority; despite publishing only 18 relevant articles, its high citation count (4,444) and IF (7.2) confirm its high average impact and academic quality. A noteworthy phenomenon is the high citation ranking of top-tier specialty journals with few relevant publications, such as *Annals of the Rheumatic Diseases* (IF 20.3, 1 article) and *Biomaterials* (IF 12.8, 10 articles). This indicates that research on PRP for OA is of sufficient quality to reach premier international journals and attract widespread attention. It also underscores the field’s significant interdisciplinary character and frontier value, with findings that have implications for the broader fields of medicine and biomaterials science.

### 4.2 The evolution and reassessment of PRP for OA research

Citation burst analysis shows that two foundational articles sparked early optimism for using PRP to treat OA. First, a pivotal 2011 comparative study in *Arthroscopy* by Kon et al. demonstrated that PRP provided more durable improvements in symptoms and function than hyaluronic acid (30). Subsequently, a high-quality, double-blind randomized controlled trial by Patel et al. in *The American Journal of Sports Medicine* (2013) provided crucial evidence-based support by showing that PRP’s efficacy significantly surpassed the placebo effect (31).

As research deepened, the academic focus shifted from if PRP is effective to how it works and which PRP is most effective. A 2020 review by Everts et al. in the *International Journal of Molecular Sciences* epitomizes this transition. It argued that therapeutic outcomes are not solely due to growth factors but are also influenced by other components like leukocytes, plasma proteins, and the patient’s individual hematological state (32). Furthermore, the review emphasized that the immense heterogeneity of PRP preparations (due to variations in platelet concentration, leukocyte content, and activation methods) is a key reason for inconsistent clinical results (33). The understanding of leukocytes in PRP is evolving. While traditional views implicated neutrophils in negative inflammatory effects, new perspectives argue that specific leukocyte components are crucial for therapeutic efficacy (34). For instance, monocyte-macrophages play a central role in immunomodulation and repair through their plasticity, polarizing toward either pro-inflammatory (M1) or pro-reparative (M2) phenotypes based on microenvironmental cues (35). Additionally, serotonin (5-HT) stored in platelet granules is now recognized as a potent immunomodulator and analgesic. Upon PRP activation, released 5-HT acts on immune cells and nerve endings to regulate inflammation and produce direct analgesic effects, providing a new biological basis for PRP’s rapid pain relief (36). The citation burst of this article signifies the field’s growing maturity, highlighting that “standardization” is the core bottleneck hindering the technology’s development. It also pinpointed “micro-mechanisms” and “personalized therapy” as inevitable future research directions.

In recent years, three high-burst articles have introduced a more cautious and critical academic attitude toward PRP for OA. First, a large, high-quality randomized controlled trial in *JAMA* (2021) by Bennell et al. challenged the field. It found no significant difference between PRP and placebo for improving knee pain or cartilage loss over 12 months, suggesting the perceived benefits may be due to a strong placebo effect rather than a specific therapeutic action (37). Second, the official 2020 clinical guidelines from the American College of Rheumatology (Kolasinski et al.) recommended against the use of PRP for treating knee and hip OA. They cited the immense “heterogeneity” of PRP preparations due to a lack of standards as the core dilemma hindering its clinical promotion (38). Lastly, even the field’s most prolific scholar, Giuseppe Filardo, in a 2021 meta-analysis, pointed out a critical nuance. He concluded that while PRP’s efficacy is statistically superior to placebo and hyaluronic acid, the advantage does not meet the standard for “minimum clinically important difference (MCID).” He also emphasized the low quality of existing evidence, calling for more definitive research (39).

Despite promising clinical outcomes, the true efficacy of PRP remains debated due to formulation heterogeneity, particularly regarding platelet concentration (40, 41). Initially, clinical evidence strongly advocated for higher doses: a recent meta-analysis showed high-concentration PRP ( > 1,000,000 platelets/μL) consistently exceeds the MCID for pain relief, while a large cohort study demonstrated that hyper-concentrated PRP ( > 1,200,000 platelets/μL) dramatically reduced treatment failure rates to 3.3% (21, 42). However, a subsequent meta-analysis challenged this linear dose-response assumption, revealing that a moderate 2–4-fold increase in baseline platelet concentration achieves maximum clinical benefits, whereas excessively high doses ( > 4-fold) paradoxically attenuate therapeutic efficacy (43). This clinical observation is elegantly corroborated by an *in vitro* model demonstrating that while low-dose PRP formulations (e.g., 75,000 platelets/μL) effectively suppress catabolic enzymes, hyper-concentrated doses (1,500,000 platelets/μL) paradoxically lose this cartilage-protective ability (44). These contradictory findings underscore the critical need to standardize protocols and identify the precise optimal therapeutic window—a targeted concentration that maximizes joint repair without inducing cellular toxicity.

### 4.3 Analysis of research hotspots and frontiers

An analysis of the keywords from the red cluster (cellular and molecular level) and the green cluster shows that “chondrocytes,” “exosomes,” and “growth-factor-beta” are the primary research targets in this field, while “inflammation,” “cartilage repair,” “cartilage defects,” and “chondrogenesis” are the main pathological states and biological processes studied. There is a growing consensus that OA is a progressive, chronic inflammatory disease. Accordingly, research now focuses not just on pathological signs like cartilage defects, but on understanding how PRP modulates these complex pathophysiological processes to achieve tissue repair. In an OA joint, synoviocytes and chondrocytes produce pro-inflammatory cytokines such as interleukin-1β (IL-1β) and tumor necrosis factor-α (TNF-α). These factors then induce matrix metalloproteinases (MMPs), which accelerate cartilage degradation and inhibit anabolic metabolism, creating a vicious pro-catabolic cycle (45). PRP appears to improve OA symptoms through its anti-inflammatory effects. Clinical evidence from a trial by Cole et al. supports this, as they found that PRP treatment decreased the levels of pro-inflammatory cytokines IL-1β and TNF-α in patients’ synovial fluid (46). Mechanistically, components within PRP, such as hepatocyte growth factor, can inhibit the NF-κB inflammatory pathway, thereby reducing the secretion of cartilage-degrading MMPs (47). Chondrocytes are the sole cell type in articular cartilage, responsible for matrix synthesis and maintenance (48). During the OA disease process, harmful stimuli like inflammation and oxidative stress cause chondrocyte apoptosis. This loss of functional cells prevents the cartilage from repairing itself, leading to irreversible structural damage (49). Research shows that PRP can counteract this damage. A study by Kruger et al. demonstrated that PRP can stimulate progenitor cells from subchondral bone to form new cartilage matrix (50). Additionally, Yang et al. found that PRP directly protects existing chondrocytes by inhibiting their apoptosis and downregulating the key catabolic factor (51). Exosomes are nano-scale membrane vesicles that, in PRP, are primarily derived from activated platelets (52). Within the joint, these exosomes deliver their contents to target cells like chondrocytes and synoviocytes, thereby exerting therapeutic effects such as anti-inflammation (53). Research by Zhang et al. demonstrated exosomes’ dual benefits: they promoted the proliferation and differentiation of chondrocytes and stem cells *in vitro* while also suppressing chondrocyte apoptosis in animal models (54). Further supporting this, a study by Liu et al. identified a specific mechanism, showing that exosomes protect chondrocytes by regulating the Wnt/β-catenin signaling pathway. More crucially, they found the therapeutic effect of exosomes in a rabbit OA model was even superior to that of activated PRP itself (55). Transforming growth factor-beta (TGF-β) is an abundant and crucial growth factor in PRP, essential for cartilage homeostasis and repair (56). It has multiple therapeutic actions: it stimulates chondrocyte proliferation and extracellular matrix synthesis, inhibits catabolic enzymes like MMPs, and reduces inflammatory factors produced by synovial fibroblasts (57–59). The pathway’s importance is highlighted by a study from Ge et al., which found that inhibiting negative regulators of the TGF-β/Smad2 signaling pathway significantly delays OA progression (60).

Insights from the citation burst analysis and the keywords from the blue cluster indicate that for the past two decades, a primary research focus has been evaluating the relative advantages of PRP by comparing it with other intra-articular injection therapies. Concurrently, exploring combination therapies with other biological agents to achieve synergistic effects is also a key research hotspot. A review by Soufan et al. highlighted that PRP offers superior and more durable pain relief and functional improvement than hyaluronic acid. The same review noted that while both improve symptoms, mesenchymal stem cells may provide greater structural benefits to the knee joint (61). Furthermore, meta-analyses have shown that a PRP and hyaluronic acid combination offers comprehensive advantages (62), and that both PRP and bone marrow aspirate concentrate are clinically superior to hyaluronic acid alone (63).

As represented by the keywords in the pink cluster, clinical trials evaluating PRP’s efficacy rely on scientific research design and evaluation standards. The formation of this cluster underscores the importance of evidence-based medicine in this field. The high frequency of terms like “double-blind” and “randomized controlled trial” indicates a growing emphasis on obtaining reliable evidence through high-quality clinical trials to assess PRP’s true efficacy. At the same time, keywords such as “follow-up,” “clinical-outcomes,” and “chronic pain” reflect a focus on long-term efficacy and patient-reported outcomes. In OA clinical studies, standardized metrics are crucial for evaluation, with pain relief being a core criterion. A prime example is the WOMAC (Western Ontario and McMaster Universities Osteoarthritis Index), an internationally used scale for knee and hip OA that served as the primary outcome measure in a prominent *New England Journal of Medicine* trial (64). The WOMAC assesses three key dimensions (pain, stiffness, and physical function) with its first and most important component being the “Pain Subscale,” reinforcing the focus on pain as a primary endpoint (65, 66).

According to the trend topics analysis, “Macrophage polarization” and “exercise” have emerged as a key research hotspot. A recent study demonstrated that PRP can promote the shift of pro-inflammatory M1-type macrophages to anti-inflammatory M2-type macrophages by inhibiting the NF-κB signaling pathway. This transformation reduces the release of pain-causing inflammatory factors, thereby alleviating OA-related inflammation and pain (67). A key study by Baria et al. found that just 4 min of high-intensity interval exercise performed before PRP preparation significantly increased platelet concentration. More critically, the concentration of cartilage-repairing TGF-β soared by over 160%. This provides new empirical evidence for optimizing PRP’s bioactivity through exercise to enhance its efficacy for osteoarthritis (68).

### 4.4 Limitation

While this study provides a systematic analysis, it has several limitations. The primary limitation concerns data coverage: although we employed a dual-database strategy, the bibliometric network visualization was based on WoSCC data due to its comprehensive citation metadata. While we supplemented this with PubMed for clinical trial analysis, literature from databases like Scopus may still have been excluded. Additionally, the restriction to English-language publications and potential author name ambiguities constitute methodological constraints. Furthermore, a limitation exists regarding specific anatomical sites. Due to the inability of bibliometric software to extract granular clinical details and the anatomical overlap in many studies (which often explore multiple joints), a site-specific analysis of PRP efficacy was unfeasible. Nevertheless, our keyword analysis unequivocally demonstrates that PRP for knee osteoarthritis remains the predominant focus of current research. Despite these factors, this study offers a robust and valuable overview of the research landscape and key trends.

## 5 Conclusion

Over the past two decades, research on PRP for OA has grown significantly, evolving from efficacy studies to exploring deep biological mechanisms like inflammation regulation and tissue regeneration. The field is now advancing toward cutting-edge frontiers such as macrophage polarization and combined exercise therapy. Current research hotspots focus on mechanistic pathways involving exosomes and TGF-β, while clinical trials continue to compare PRP against agents like hyaluronic acid, mesenchymal stem cells, and corticosteroids. However, the field faces significant challenges: the clinical benefits of PRP are being questioned by recent high-quality trials, and the lack of standardization for PRP preparations remains a major hurdle for its broader clinical translation. In summary, the field of PRP for OA is at a critical crossroads, balancing the immense potential of regenerative medicine with the urgent need for high-quality, standardized evidence. Future research must prioritize standardizing PRP preparation protocols and conducting in-depth explorations of its immunomodulatory mechanisms, while ultimately launching more rigorously designed, large-scale clinical trials to definitively clarify its value and position in treating OA. This study provides a comprehensive roadmap, offering clear insights into the past, present, and future of this complex and promising medical domain.

## Data Availability

The original contributions presented in this study are included in the article/supplementary material, further inquiries can be directed to the corresponding author.
